# miR‐124 interacts with the Notch1 signalling pathway and has therapeutic potential against gastric cancer

**DOI:** 10.1111/jcmm.12724

**Published:** 2015-11-27

**Authors:** Lei Jiang, Tiesu Lin, Chaochao Xu, Sunkuan Hu, Yangyang Pan, Rong Jin

**Affiliations:** ^1^ Central LaboratoryThe First Affiliated Hospital of Wenzhou Medical UniversityWenzhouZhejiangChina; ^2^ Department of GastroenterologyThe First Affiliated Hospital of Wenzhou Medical UniversityWenzhouZhejiangChina; ^3^ Department of EpidemiologyThe First Affiliated Hospital of Wenzhou Medical UniversityWenzhouZhejiangChina

**Keywords:** miR‐124, Notch1, JAG1, intracellular domain of Notch1, gastric cancer

## Abstract

Aberrant Notch signalling plays an important role in cancer progression. However, little is known about the interaction between miRNA and the Notch signalling pathway and its role in gastric cancer (GC). In this study, we found that miR‐124 was down‐regulated in GC compared with adjacent normal tissue. Forced expression of miR‐124 inhibited GC cell growth, migration and invasion, and induced cell cycle arrest. miR‐124 negatively regulated Notch1 signalling by targeting JAG1. miR‐124 levels were also shown to be inversely correlated with JAG1 expression in GC. Furthermore, we found that the overexpression of the intracellular domain of Notch1 repressed miR‐124 expression, promoted GC cell growth, migration and invasion. Conversely, blocking Notch1 using a γ‐secretase inhibitor up‐regulated miR‐124 expression, inhibited GC cell growth, migration and invasion. In conclusion, our data demonstrates a regulatory feedback loop between miR‐124 and Notch1 signalling in GC cells, suggesting that the miR‐124/Notch axis may be a potential therapeutic target against GC.

## Introduction

Gastric cancer (GC) is one of the most common malignant tumours in the world and more than 50% of cases occur in East Asia where GC is the second leading cause of cancer death 1. Despite the significant achievements in the treatment of early GC, the prognosis for advanced GC is still poor. Hence, it is urgent to identify key molecules during GC development and progression to optimize therapeutic options.

Micro‐RNAs (miRNAs) are a class of diverse, small, non‐coding RNAs that negatively regulate protein expression through specifically binding to the 3′ untranslated region (UTR) of target mRNAs. Micro‐RNAs regulate gene expression post‐transcriptionally and can either degrade target mRNAs or impair their translation 2. Micro‐RNAs play an important role in various biological processes, including cell proliferation, differentiation, growth and death 3. In recent years, a number of studies have revealed that the deregulation of miRNAs is involved with cancer development and progression 4, 5, suggesting that miRNAs may present as a novel class of oncogene or tumour suppressor gene 6.

miR‐124 is a brain‐enriched miRNA that plays an important role in neuronal development 7. Recent studies have shown that miR‐124 was down‐regulated in various cancers including glioma 8, breast cancer 9, non‐small‐cell lung cancer 10, hepatocellular carcinoma 11, GC 12 and nasopharyngeal carcinoma 13. miR‐124 also inhibits GC cell proliferation by targeting SPHK1 12. Additionally, ectopic expression of miR‐124 suppresses GC cell proliferation, migration and invasion through down‐regulation of ROCK1 14. miR‐124 was also reported to inhibit proliferation and induce apoptosis by targeting EZH2 in GC 15. The Notch signalling plays an important role in cell differentiation, survival and proliferation 16. Aberrant Notch signalling can often lead to the development of cancer. Our previous work showed that Notch1 intracellular domain (NICD) protein is overexpressed in GC tissues and is associated with overall patient survival, tumour metastasis and an advanced tumour stage 17. In fact, expression of Notch1 and Hes1 was significantly higher in GC tissues than in normal tissues 18. These results suggest that Notch1 signal pathway plays an important role in GC progression. However, little is known about the interaction between miRNAs and the Notch signalling pathway in GC.

In this study, we found that miR‐124 was down‐regulated in GC specimens and cell lines. Ectopic expression of miR‐124 inhibited GC cell growth, migration and invasion, induced cell cycle arrest. In addition, miR‐124 can regulate Notch1 signalling by targeting Notch ligand Jagged1 (JAG1) in GC cells. Furthermore, Notch activation by ectopic expression of the NICD showed repression of miR‐124 expression, and the promotion of GC cell growth, migration and invasion. Conversely, we used a γ‐secretase inhibitor to block Notch1 signalling which resulted in the up‐regulation of miR‐124 expression, and the inhibition of GC cell growth, migration and invasion. Our data provides additional evidence of a pivotal role for miR‐124 in GC, and suggests a regulatory feedback loop between miR‐124 and the Notch1 signalling pathway in GC cells.

## Material and methods

### Cell culture and tissue samples

Human gastric epithelial immortalized GES‐1 cell line and GC cell lines SGC‐7901, BGC‐823, MGC‐803, KATO‐3 and AGS were purchased from the Type Culture Collection of the Chinese Academy of Sciences, Shanghai, China. All cells were cultured in RPMI 1640 medium or Dulbecco's modified Eagle medium supplemented with 10% foetal bovine serum and 100 U/ml penicillin/streptomycin (Invitrogen, Carlsbad, CA, USA) in a humidified incubator at 37°C with 5% CO_2_. Eight cases of fresh GC tissues and paired adjacent non‐tumour tissues (at least 5 cm away from the tumour margin) were collected with full informed consent from patients (five male and three female patients aged 38–67 years) at The First Affiliated Hospital of Wenzhou Medical University. All patients had not received radiotherapy or chemotherapy prior to surgery. The tissues were verified by a trained pathologist. According to Lauren's criteria, three cases were of intestinal type and five cases were of diffuse type. By pathological grading, two cases were well differentiated, four moderately differentiated and two poorly differentiated. According to the WHO histological classification, one case was papillary adenocarcinoma, three cases were tubular adenocarcinoma, two were mucinous adenocarcinoma and two were signet‐ring cell carcinoma. Two cases were in TNM stage I, four in TNM stage II and two in TNM stage III. One case had lymph node metastases. Tissue samples were immediately snap‐frozen in liquid nitrogen and stored at −80°C till isolation of RNA and protein. The study was approved by the Ethics Committee of Wenzhou Medical University.

### miRNA mimics and inhibitors transfection

The has‐miR‐124 mimics, has‐miR‐124 inhibitors and negative control (NC) oligonucleotides were purchased from GenePharma. miR‐124 mimics were transfected into the SGC‐7901 and BGC‐823 cells, which had a relatively low expression level of miR‐124 compared with the normal gastric cell line GES‐1. miR‐124 inhibitors were transfected into GES‐1, which had a relatively high expression level of miR‐124 compared with GC cells. Transfection of cells with oligonucleotides was performed with Lipofectamine RNAiMAX reagent (Invitrogen) at a final concentration of 100 nM has‐miR‐124 mimics or 200 nM has‐miR‐124 inhibitors according to the manufacturer's instructions.

### Reverse transcription‐PCR

Total RNA was extracted from tissue samples and cells using Trizol (Invitrogen) according to the manufacturer's instructions. Micro‐RNA reverse transcription (RT) reactions were performed with stem‐loop primers (Table 1). The expression levels of miR‐124 were evaluated by quantitative real‐time PCR. The U6 small nuclear RNA was used for normalization. The JAG1 mRNA level was determined by real‐time PCR using SYBRs GREEN PCR Master Mix (Applied Biosystems, Foster City, CA, USA) following the manufacturer's instruction and PCR amplification was carried out in an ABI7500 Real‐time PCR system (Applied Biosystems). Glyceraldehyde 3‐phosphate dehydrogenase (GAPDH), an endogenous housekeeping gene, was used for normalization. The primers are shown in Table 1.

**Table 1:** Primers used for PCR amplification of miRNA and genes

Gene name	Sequence (5′ → 3′)	
miR‐124	RT primer	GTCGTATCCAGTGCAGGGTCCGAGGTATTCGCACTGGATACGACGGCATTCA
	Forward	ATGGTTGGTTGGTAAGGCACGCGG
	Reverse	GCAGGGTCCGAGGTATTC
U6	RT primer	AACGCTTCACGAATTTGCGT
	Forward	CTCGCTTCGGCAGCACA
	Reverse	AACGCTTCACGAATTTGCGT
JAG1	Forward	GGGGCAACACCTTCAACCTC
	Reverse	CCACGCCTCCACAAGCAAC
GAPDH	Forward	TCCCATCACCATCTTCCAGG
	Reverse	GATGACCCTTTTGGCTCCC
JAG1 3′UTR Site Awt	Forward	GGACTAGTTGCCAGATGTCCTAATGGTG
	Reverse	AGCTTTGTTTAAACCCAGCAACTGCTGACATCAA
JAG1 3′UTR Site Bwt	Forward	GGACTAGTCCATTCGTACATAATACTGAACCAC
	Reverse	AGCTTTGTTTAAACTCTTCACGGTCTCAATGGTG
JAG1 3′UTR Site Amu	Forward	CTAGTACAAGTAGTTCTGTATTTGAAAGTCCGCTCGCAGCTCAGAACCACATAAGTTT
	Reverse	AAACTTATGTGGTTCTGAGCTGCGAGCGGACTTTCAAATACAGAACTACTTGTA
JAG1 3′UTR Site Bmu	Forward	CTAGTTAGATTTGCCATAGAGTACTGTGCCTCCGCTAAGTGAGGAAATCAATAAGTTT
	Reverse	AAACTTATTGATTTCCTCACTTAGCGGAGGCACAGTACTCTATGGCAAATCTAA

### Western blotting

Cells were washed with ice‐cold PBS and lysed on ice in Radio‐Immunoprecipitation Assay (RIPA) buffer (Sigma‐Aldrich, St. Louis, MO, USA) with Protease Inhibitor Cocktail (Sigma‐Aldrich). For preparation of lysate from tissues, frozen tissues were sliced using a clean razor blade and thawed in RIPA buffer containing protease Inhibitors. Equal amounts of proteins (30 μg) were loaded into the wells of the SDS‐PAGE gel and then transferred onto polyvinylidene fluoride membranes (Millipore, Bedford, MA, USA). The membranes were blocked with 5% non‐fat milk in TBST for 1 hr. After blocking, the membranes were incubated with the respective primary antibodies overnight at 4°C. The primary antibodies used were as follows: rabbit anti‐GAPDH antibody (Ab; Santa Cruz Biotechnology, Santa Cruz, CA, USA), or rabbit anti‐JAG1 Ab (Santa Cruz Biotechnology), mouse anti‐HES1 Ab (Santa Cruz Biotechnology), rabbit anti‐HES5 Ab (Abcam, Cambridge, UK) and rabbit anti‐NICD Ab (Millipore). After washing, membranes were incubated in PBS with 0.1% Tween with antimouse or anti‐rabbit IgG secondary antibody conjugated with horseradish peroxidase (Santa Cruz Biotechnology) for 1 hr. Blots were detected using enhanced chemiluminescence substrate kit (GE Healthcare, Piscataway, NJ, USA).

### Cell proliferation assay

Cell proliferation was determined using the Cell Counting Kit‐8 (CCK‐8) assay (Dojindo Co. Ltd., Kumamoto, Japan). Briefly, 5000 cells (100 μl/well) were seeded on 96‐well plates. At indicated time‐points, 10 μl of the CCK‐8 solution was added to each well, respectively, then incubated for another 2 hrs at 37°C. After incubation, the corresponding absorbance was measured at 450 nm using a microplate reader.

### Cell cycle analysis

Cell cycle analysis was performed with propidium iodide (Sigma‐Aldrich) staining as described previously 19. Briefly, 2 × 10^5^ cells per well in 6‐well plates were transfected with 100 nM has‐miR‐124 mimics for 48 hrs. Cells were collected and fixed in 70% (v/v) ethanol on ice. Cells were then washed with PBS and suspended in propidium iodide staining solution (50 mg/l) that contains 0.1% Triton X‐100 and RNase (100 mg/l). After 30 min. of incubation, cell fluorescence was measured using a FACS Calibur flow cytometer (BD Biosciences, San Jose, CA, USA), and cell cycle analysis was performed with the ModFit LT 2.0 software.

### Migration and invasion assay

Cell migration and invasion were analysed using a transwell assay. Costar transwell cell culture chamber inserts (8‐μm pore size membranes; Corning, Cambridge, MA, USA) were used. For the migration assay, 5 × 10^4^ cells were seeded in the upper chamber in 100 μl of serum‐free medium fetal bovine serum (FBS) (10%) was added to the lower chamber as a chemoattractant. After 24 hrs incubation, non‐migratory cells in the upper chamber were removed and the cells that migrated to the lower chamber were stained and quantified. The cells were counted under a microscope in five randomly selected fields. To analyse invasion, the upper surface of the insert membrane was first coated with 1:3 diluted matrigel (BD Biosciences, San Jose, CA, USA) and the subsequent procedures were the same as the migration assay. The relative cell migration and invasion activity was calculated after normalization to the mock group. All experiments were performed in triplicates.

### Plasmid transfection and luciferase reporter assay

The plasmid, pIRES2‐EGFP, containing the NICD cDNA (pIRES‐NICD) was kindly gifted by Dr. Zheng 20. pIRES‐NICD was transfected into AGS cells, which had a relatively low expression level of Notch1 mRNA compared with other GC cells. To construct reporter plasmids, two DNA fragments of the JAG1 3′UTR containing the putative miR‐124 binding sites A and B were amplified from human genomic DNA and cloned into the Pme I ‐ Spe I site of the pMIR‐REPORT luciferase vector (Ambion, Carlsbad, CA, USA). Primers for wild‐type JAG1 3′UTR sites A and B and mutation sites A and B are showed Table 1. DNA oligonucleotides for JAG1 3′UTR mutation sites A and B were annealed and cloned into the pMIR‐REPORT luciferase vector. The sequences were confirmed by DNA sequencing analysis. For the luciferase assay, 4 × 10^4^ SGC‐7901 cells per well were seeded onto 24‐well plates. After incubation for 24 hrs, the luciferase reporter construct was cotransfected with 100 nM miR‐124 mimics or miRNA‐NC (miR‐NC). The pRL‐SV40 Renilla luciferase plasmid (Promega, Madison, WI, USA) was used as an internal control. Two days after transfection, cells were lysed and assayed for luciferase activity using a dual luciferase reporter assay (Promega).

### Statistical analysis

All experiments were performed in triplicate, and data are expressed as mean ± S.E. Statistical analysis was conducted using the independent samples *t*‐test or one‐way anova with SPSS 13.0 (IBM, Armonk, NY, USA). *P*‐values of less than 0.05 were considered statistically significant.

## Results

### Overexpression of miR‐124 inhibits GC cell growth, migration and invasion and induces cell cycle arrest

**Figure 1:**
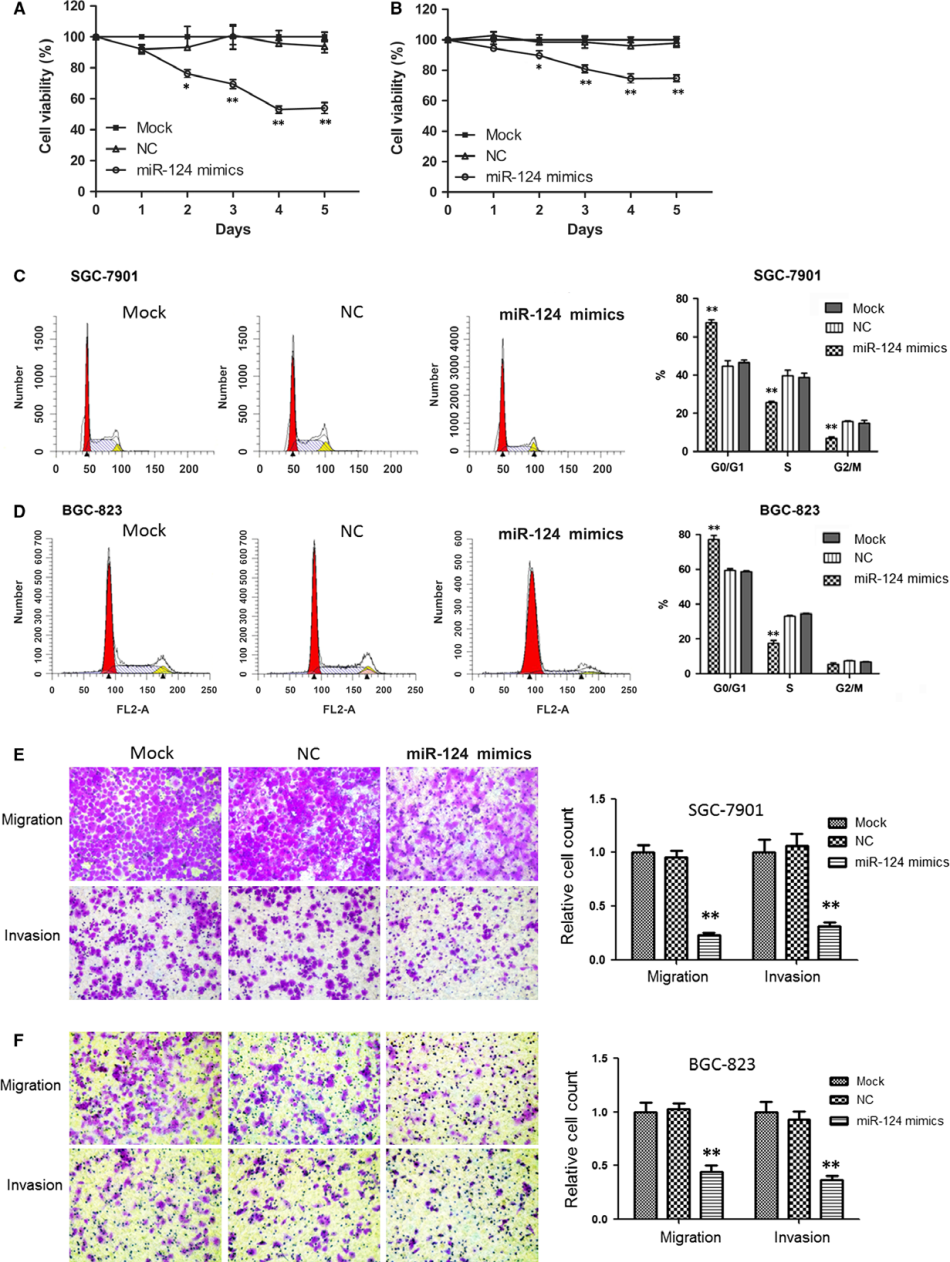
Overexpression of miR‐124‐inhibited GC cell growth, migration and invasion, and induced cell cycle arrest. (**A** and **B**) Overexpression of miR‐124 caused a significant growth inhibition of SGC‐7901 and BGC‐823 cells revealed using MTT assay. (**C** and **D**) SGC‐7901 and BGC‐823 cells were arrested in the G0/G1 phase of the cell cycle by miR‐124 mimics. The cell cycle distribution of SGC‐7901 and BGC‐823 cells transfected with 100 nM miR‐124 mimics and negative controls are shown. The percentages of cells in G0/G1, S and G2/M phases were quantitatively analysed. (**E** and **F**) Overexpression of miR‐124‐inhibited SGC‐7901 and BGC‐823 cell migration and invasion. Microphotographs were taken with 100× magnifications. Statistical analysis was conducted using one‐way anova, **P < 0.01 (*versus* NC group).

To evaluate the effect of miR‐124 on GC cell growth, miR‐124 mimics were transfected into SGC‐7901 and BGC‐823 cells. We found that overexpression of miR‐124 led to a significant growth inhibition of SGC‐7901 (Fig. 1A) and BGC‐823 GC cells compared with untransfected (mock) or NC groups (Fig. 1B). When transfected with miR‐124 on day 4, cell viability significantly decreased in both the SGC‐7901 (53.0%) and BGC‐823 (74.5%) cells compared to their NC group cells (95.8% and 96.0%; *P* < 0.01).

Change in the cell cycle distribution of SGC‐7901 and BGC‐823 cells transfected with miR‐124 mimics was also observed using flow cytometry. As seen in Figure 1C and D, SGC‐7901 and BGC‐823 cells were arrested in the G0/G1 phase of the cell cycle by miR‐124 transfection (*P* < 0.01). About 67.5% of SGC‐7901 cells in the miR‐124‐treated group were arrested in G0/G1, whereas 44.7% of the NC group cells and 46.5% of the mock group cells were in G0/G1. Similarly, when BGC‐823 cells were transfected with miR‐124 mimics for 48 hrs, it resulted in 77.1% of the cell population being in the G0/G1 phase (NC group: 59.5%, mock group: 58.7%). These results indicate that miR‐124 induces G0/G1 phase arrest in GC cells.

The effects of miR‐124 on migration and invasion of GC cells were evaluated using the Transwell assay. As shown in Figure 1E and F, compared with NC and mock groups, miR‐124 significantly inhibited SGC‐7901 and BGC‐823 cell migration and invasion (*P* < 0.01). Ectopic expression of miR‐124 in SGC‐7901 and BGC‐823 cells reduced cell counts in the migration assay by 76.0% and 57.1%, respectively, and in the invasion assay by 70.9% and 60.4%, respectively.

### miR‐124 affects Notch1 signalling by targeting the JAG1 gene

**Figure 2:**
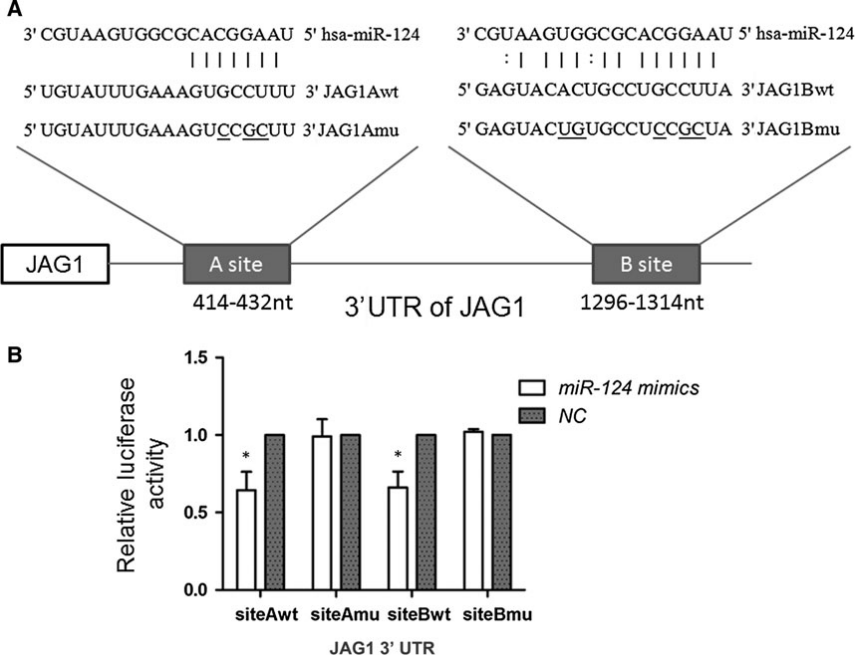
miR‐124 targeted the 3′UTR of the JAG1 gene. (**A**) Schematic of putative binding sites of miR‐124 in the JAG1 3′UTR, showing predicted pairing with two target sites and their respective mutant sequences. JAG1Amu and JAG1Bmu show JAG1 3′UTR with mutations in miR‐124 A and B binding sites (the underline indicates site of mutation). (**B**) miR‐124 mimics suppressed the activity of a luciferase reporter containing wild‐type JAG1 3′UTR, but not the reporter with mutant JAG1 3′UTR. Independent samples *t*‐test was used to analyse the difference between two groups; *P < 0.05.

To investigate whether JAG1 is a target of miR‐124, we screened the 3′UTR region of JAG1 mRNA using TargetScanHuman 6.2 (http://www.targetscan.org/). Figure 2A shows two potential binding sites of miR‐124 in the 3′UTR of JAG1. Putative binding sites of wild‐type (Luc‐siteAwt and Luc‐siteBwt) and mutant (Luc‐siteAmu and Luc‐siteBmu) were cloned into the pMIR‐REPORT luciferase reporter vector (Fig. 2A). MiR‐124 was observed to suppress luciferase reporter activity of Luc‐siteAwt and Luc‐siteBwt (*P* < 0.05), whereas no such inhibitory effect was seen on the reporters with mutant JAG1 3′UTR (Luc‐siteAmu and Luc‐siteBmu; Fig. 2B).

**Figure 3:**
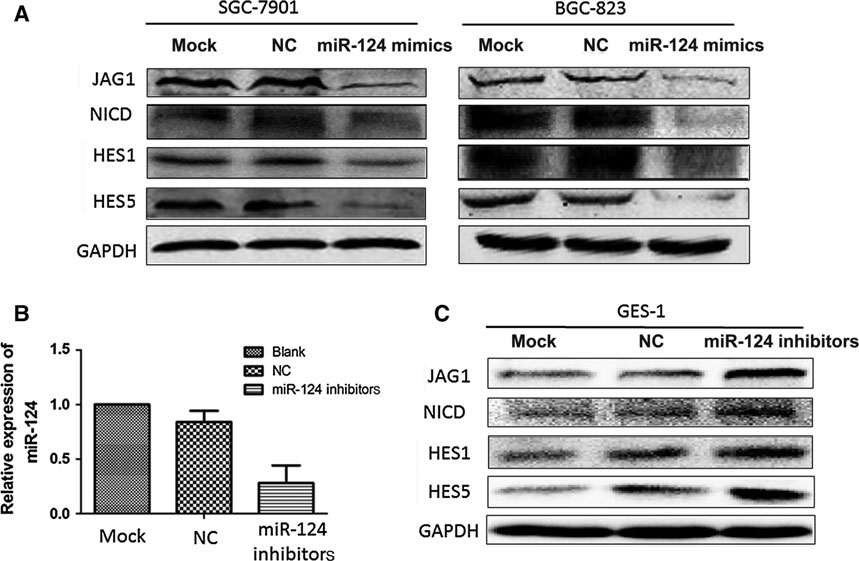
miR‐124 affects the Notch signalling pathway by targeting JAG1. (**A**) Overexpression of miR‐124 caused significant reduction in the expressions of the Notch‐1 ligand (JAG‐1), cleaved Notch‐1 (NICD), and Notch‐1 target genes HES‐1 and HES‐5 in both SGC‐7901 and BGC‐823 cells. (**B**) GES‐1 cells were transfected with miR‐124 inhibitors and the miR‐124 expression was determined using real‐time PCR. (**C**) Transfection of miR‐124 inhibitors up‐regulated the expressions of JAG1, NICD, HES1 and HES5 in GES‐1 cells.

To assess the effects of miR‐124 on JAG1 expression, miR‐124 mimics were transfected into the SGC‐7901 and BGC‐823 cell lines, which had a relatively low expression level of miR‐124 compared with the human gastric epithelial immortalized GES‐1 cell line. JAG1, NICD and Notch effectors (HES1 and HES5) protein levels were determined by Western blot analysis. Figure 3A shows miR‐124 inhibiting the expression of JAG1 as well as the Notch1 signalling pathway. Conversely, the inhibition of miR‐124 showed an elevated JAG1 expression level as well as an increase in the Notch1 signalling pathway (Fig. 3B and C). These results suggest that miR‐124 targeted the 3′UTR region of JAG1 and inhibited JAG1 expression, thereby negatively regulating the Notch1 signalling pathway.

### JAG1 expression correlates with miR‐124 expression in clinical specimens and cell lines

**Figure 4:**
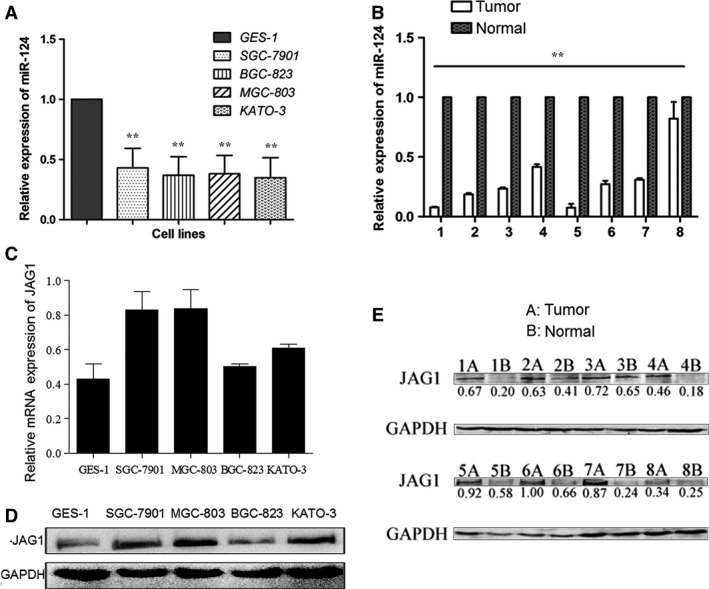
Negative correlation between miR‐124 and JAG1 expression in clinical specimens and cell lines. (**A**) The levels of miR‐124 expression were determined in human gastric epithelial immortalized GES‐1 cell line and GC cell lines (SGC‐7901, BGC‐823, MGC‐803, KATO‐3) using qRT‐PCR. The relative miR‐124 expression in gastric cancer cell lines was much lower than that of GES‐1. One‐way anova test was conducted. (**B**) The levels of miR‐124 expression were determined in eight cases of fresh GC tissues and paired adjacent non‐tumour tissues using qRT‐PCR. Independent samples *t*‐test was used to analyse the difference between two groups. (**C**) The levels of JAG1 mRNA expression were determined in GES‐1, SGC‐7901, BGC‐823, MGC‐803 and KATO‐3 cells using qRT‐PCR. (**D**) Western blot analyses of JAG1 protein levels in GES‐1 and four cancer cell lines. (**E**) Western blot analyses of JAG1 protein levels in GC tissues and paired adjacent non‐tumour tissues. Densitometric quantification of JAG1 band intensities were normalized to GAPDH, ratios are shown under the bands. Expression of miR‐124 and JAG1 exhibited a significant inverse correlation calculated by Pearson's correlation (*r* = −0.67, *P* < 0.05). **P < 0.01.

To determine the expression levels of JAG1 and miR‐124 in gastric carcinoma, we selected eight pairs of GC tissues and matched normal tissues adjacent to the tumour, and one normal gastric cell line GES‐1 and four malignant human GC cell lines (SGC‐7901, MGC‐803, BGC‐823 and KATO‐3). The levels of miR‐124 and JAG1 mRNA were detected using qRT‐PCR, and the levels of the JAG1 protein were confirmed by Western blot analysis. As shown in Figure 4A and B, miR‐124 was down‐regulated in cancer tissues (compared to normal tissues, *P* < 0.01) and GC cell lines (compared to GES‐1 cells, *P* < 0.01). On the other hand, the JAG1 mRNA expression was up‐regulated in GC cell lines (Fig. 4C) and protein expression was up‐regulated in human GC samples and GC cell lines (Fig. 4D and E). Expression of miR‐124 and JAG1 exhibited a significant inverse correlation calculated by Pearson's correlation (*r* = −0.67, *P* < 0.05).

### NICD negatively regulates miR‐124 expression, promotes cell growth, migration and invasion of GC cells

**Figure 5:**
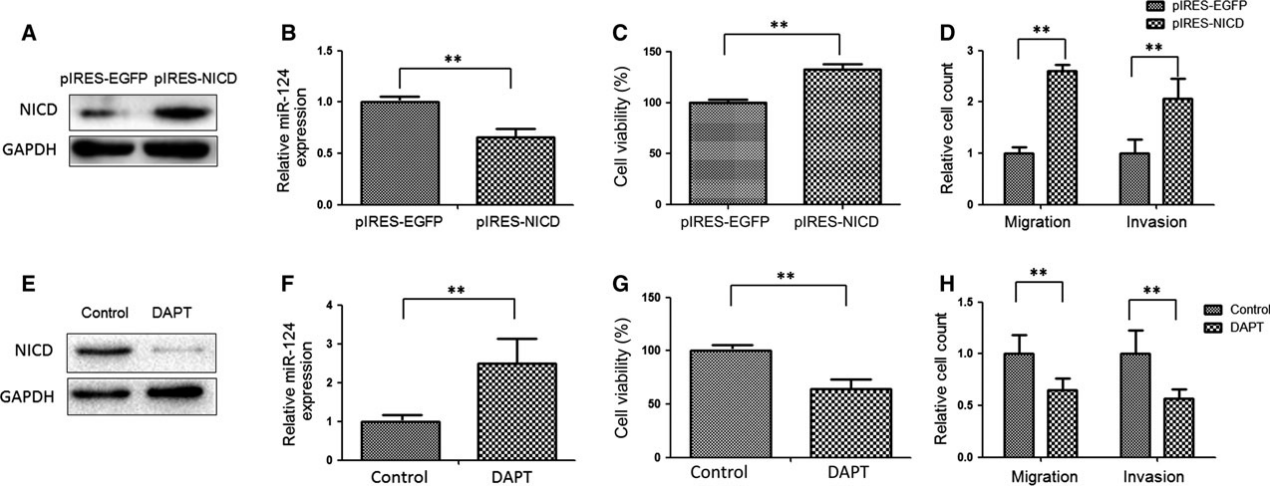
NICD negatively regulates miR‐124 expression, promotes cell growth, migration and invasion in GC cells. (**A**) Immunoblot analysis of cleaved Notch‐1 (NICD) in AGS transfected for 48 hrs with pIRES‐ NICD or control pIRES‐EGFP vector. (**B**) Overexpression of NICD down‐regulated miR‐124 expression. (**C**) Overexpression of NICD promoted AGS cell growth. AGS were transfected with pIRES‐NICD or control pIRES‐EGFP vector. Cell viability was measured using CCK‐8 assay at 72 hrs. (**D**) Overexpression of NICD promoted AGS cell migration and invasion. (**E**) Immunoblot analysis of NICD in BGC‐823 cells treated for 48 hrs with DAPT. (**F**) Real‐time PCR analysis of miR‐124 expression in BGC‐823 cells treated for 48 hrs with DAPT. (**G**) Inhibition of Notch‐1 signalling using DAPT suppressed cell growth. Cell viability was measured 48 hrs after DAPT treatment using CCK‐8 assay. (**H**) Inhibition of Notch‐1 signalling suppressed BGC‐823 cell migration and invasion. Independent samples *t*‐test was used to analyse the difference between two groups; **P < 0.01.

To explore the role of Notch1 signalling in GC, the Notch1 expression levels of five GC cell lines (SGC‐7901, BGC‐823, MGC‐803, KATO‐3 and AGS) were determined using real‐time PCR. BGC‐823 cells had a relatively high level of Notch1 mRNA, whereas AGS cells had a relatively low level of Notch1 mRNA compared with other GC cells (data not shown). AGS cells were selected to be transfected with pIRES‐NICD and BGC‐823 cells were treated with the Notch signalling inhibitor DAPT (N‐(3,5‐Difluorophenacetyl)‐L‐alanyl]‐S‐phenylglycine t‐butyl ester). Overexpression of NICD in AGS cells transfected with pIRES‐NICD was confirmed by Western blot analysis (Fig. 5A). Real‐time quantitative RT‐PCR showed that the overexpression of NICD resulted in significant decreases in miR‐124 expression level *versus* cells transfected with pIRES‐EGFP at 48 hrs (Fig. 5B, *P* < 0.01). In addition, we found that the overexpression of NICD was accompanied by a substantial increase in cell growth, migration and invasion of AGS cells *in vitro* (Fig. 5C and D). The pIRES‐NICD transfection resulted in a 31.4% increase in cell viability compared with pIRES‐EGFP transfection in GC cells (*P* < 0.01). The number of migration and invasion of NICD‐expressioned cells is 2.63 and 2.05‐fold higher compared to that of EGFP‐expression cells (*P* < 0.01).

DAPT, an inhibitor of γ‐secretase, can inhibit Notch signalling activation. As shown as Figure 5E, Western blot analysis showed that 20 μM DAPT treatment for 48 hrs inhibited NICD protein expression in BGC‐823 cells. Real‐time PCR analysis showed that DAPT treatment increased miR‐124 expression in BGC‐823 cells (Fig. 5F; *P* < 0.01). Moreover, blocking Notch1 signalling using DAPT significantly decreased GC cell viability by 33.1% at 48 hrs (Fig. 5G). We also tested the effect of DAPT on BGC‐823 at 24 hrs and found that DAPT treatment had a little effect on cell growth at 24 hrs (about 10% mean reduction in cell growth, data not shown). However, blocking Notch1 signalling using DAPT significantly decreased GC cell migration and invasion by 35.6% and 41.1% at 24 hrs (Fig. 5H; *P* < 0.01).

## Discussion

Increasing evidence demonstrated that dysregulated miRNA expression was detected in many types of human cancer, and miRNAs can act either as potential oncogenes or tumour suppressor genes through their interactions with tumour‐related genes 6, 21. Recently, dysregulated miR‐124 expression and suppressive effects of miR‐124 on tumour growth and metastasis were reported in several cancers 8, 9, 10, 11, 12, 13, 14, 15, 22, 23, 24, 25, 26, 27, 28, 29, 30, 31, 32, 33. In line with previous studies, our data show that miR‐124 is down‐regulated in GC cell lines and cancer samples. The ectopic expression of miR‐124 suppressed GC cell proliferation, migration and invasion, and induced cell cycle arrest *in vitro*. Moreover, JAG1, a ligand of Notch1 signalling, was further verified as a direct target of miR‐124 in GC using luciferase reporter assays and Western blot. Importantly, the expression of miR‐124 was inversely correlated with JAG1 protein expression level in GC. miR‐124 was also found to negatively regulate Notch1 signalling by targeting JAG1 through binding to the 3′UTR of JAG1 and is in turn inhibited by Notch1.

The Notch signalling pathway plays an important role in cell proliferation, survival, apoptosis and differentiation 34. Notch can be activated by interacting with its cell‐bound ligands 34. To date, five members of notch ligands have been found in mammals: DLL‐1, DLL‐3, DLL‐4, JAG1 and JAG2 34. In the canonical Notch signalling pathway, Notch signalling is established when a Notch ligand expressed on the cell surface, interacts with a Notch receptor expressed on the surface of a neighbouring cell. Upon activation, Notch is sequentially cleaved to release NICD, which translocate into the nucleus and binds to the transcription factor CSL to form a transcriptional activation complex that increases expression of the bHLH transcription factors (Hes and Hey families) 35. Aberrantly activated Notch signalling has been observed in a wide range of human cancers 36. The association between the Notch signalling pathway and GC was studied by a meta‐analysis which included 15 studies with a total of 1547 GC cases and 450 controls 18. Notch1 and Hes1 expression levels were significantly higher in GC compared to normal tissues. Stratified analyses showed that JAG1 was significantly overexpressed in diffuse type and poor differentiation type of GC. Furthermore, JAG1 expression was also correlated with aggressiveness of GC and a poor survival rate. The activated form of the Notch1 receptor, NICD, promoted tumour growth, colony formation, and migration and invasion of GC SC‐M1 cells 37. Our results show that inhibition of Notch1 signalling by DAPT treatment inhibited GC cell growth, migration and invasion, whereas overexpression of NICD promoted GC cell growth, migration and invasion.

Recently, miRNAs have been reported to crosstalk with the Notch signalling pathway 16, 34. Several miRNAs were shown to regulate the Notch signalling pathway by targeting Notch ligands. For example, miR‐1 and miR‐34a were reported to target the Notch ligand DLL1 38, 39 where protein levels were down‐regulated by miR‐1 in mouse embryonic stem cells 39. miR‐34a also negatively regulates cell proliferation, and induces apoptosis and neural differentiation in medulloblastoma cells by targeting DLL1 38. Additionally, miR‐34a has been found to inhibit cell proliferation, migration, invasion and breast cancer stem cell propagation through down‐regulating Notch1 40. The miR‐200 family can also target Notch pathway components, such JAG1, MAML2 and MAML3 41.

Our work not only indicates miR‐124's inhibition of Notch pathway members and activity but also the suppression of miR‐124 by Notch1 in GC cells. Blocking Notch1 signallling up‐regulated miR‐124 expression, whereas overexpression of NICD resulted in the repression of miR‐124 expression in GC cells. It has been reported that NF‐κB activation could down‐regulate the expression of miR‐124 in lung cancer cells 24. As Hes1, a canonical Notch1 target, is able to induce NF‐κB activation in human T‐cell leukaemia and animal models of the disease 24, we speculate that Notch1 might down‐regulate miR‐124 through activation of the NF‐κB pathway in GC cells, but this hypothesis needs further study. miR‐124 might also interact with Notch signalling to regulate the Ciona epidermal‐peripheral nervous system (PNS) fate choice in tail midline cells 42. In the Ciona, blocking Notch signalling in the epidermis was carried out by expressing a dominant‐negative form of Suppressor of Hairless, which resulted in the expansion of the PNS along the tail midlines, with a corresponding expansion of the miR‐124 expression domain. Conversely, epidermal ectopic activation of Notch signalling through the expressing NICD, eliminates tail midline epidermal sensory neurons formation, with a corresponding repression of miR‐124 expression 42. When tested in their epidermal sensor assay, five Notch pathway genes (Notch, Neuralized and the three Ciona Hes genes: Hes1, HesB and HesL) were significantly down‐regulated by miR‐124 42.

Along with recent discoveries of the profound role of miRNAs in cancer, modulating miRNA expression is a promising avenue for the development of novel therapeutics for cancer therapy 43. Two human clinical trials, anti‐miR122 therapy against chronic hepatitis C and liposome‐based mimic of miR‐34 (MRX34) against liver cancers, have assessed directed miRNA‐targeting as therapeutics 43, 44. Although several important issues including the delivery method, improved oligonucleotide modification for delivery and safety remain to be addressed, miRNA‐targeted therapy appears to hold significant promise for cancer treatment 43, 44. Comprehensive understanding of the complex miRNA gene regulatory networks in cancer will help to design combinatorial therapeutic strategies 44. However, further research is needed to translate miRNA‐based cancer therapeutics into a clinical reality.

In conclusion, our data shows miR‐124 as a tumour suppressor during GC progression, which also interacts with the Notch1 signalling pathway, thereby suggesting a miR‐124/Notch axis that may have potential therapeutic implications for GC.

## Conflicts of interest

The authors confirm that there are no conflicts of interest.
